# Anthropogenic Factors Driving Recent Range Expansion of the Malaria Vector *Anopheles stephensi*

**DOI:** 10.3389/fpubh.2019.00053

**Published:** 2019-03-14

**Authors:** Sinnathamby N. Surendran, Kokila Sivabalakrishnan, Arthiyan Sivasingham, Tibutius T. P. Jayadas, Kalingarajah Karvannan, Sharanga Santhirasegaram, Kanapathy Gajapathy, Meena Senthilnanthanan, SHP Parakrma Karunaratne, Ranjan Ramasamy

**Affiliations:** ^1^Department of Zoology, University of Jaffna, Jaffna, Sri Lanka; ^2^Department of Chemistry, University of Jaffna, Jaffna, Sri Lanka; ^3^Department of Zoology, University of Peradeniya, Peradeniya, Sri Lanka; ^4^ID-FISH Technology, Miltipas, CA, United States

**Keywords:** *Anopheles stephensi*, coastal zone, insecticide resistance, malaria, mosquito adaptation to anthropogenic habitats, mosquito range expansion, urbanization, vector biology

## Abstract

The malaria vector *Anopheles stephensi* is found in wide tracts of Asia and the Middle East. The discovery of its presence for the first time in the island of Sri Lanka in 2017, poses a threat of malaria resurgence in a country which had eliminated the disease in 2013. Morphological and genetic characterization showed that the efficient Indian urban vector form *An. stephensi sensu stricto* or *type* form, has recently expanded its range to Jaffna and Mannar in northern Sri Lanka that are in proximity to Tamil Nadu state in South India. Comparison of the DNA sequences of the *cytochrome oxidase subunit 1* gene in *An. stephensi* in Jaffna and Mannar in Sri Lanka and Tamil Nadu and Puducherry states in South India showed that a haplotype that is due to a sequence change from valine to methionine in the cytochrome oxidase subunit 1 present in the Jaffna and Mannar populations has not been documented so far in Tamil Nadu/Puducherry populations. The Jaffna *An. stephensi* were closer to Tamil Nadu/Puducherry populations and differed significantly from the Mannar populations. The genetic findings cannot differentiate between separate arrivals of the Jaffna and Mannar *An. stephensi* from Tamil Nadu or a single arrival and dispersion to the two locations accompanied by micro-evolutionary changes. *Anopheles stephensi* was observed to undergo preimaginal development in fresh and brackish water domestic wells and over ground cement water storage tanks in the coastal urban environment of Jaffna and Mannar. *Anopheles stephensi* in Jaffna was resistant to the common insecticides deltamethrin, dichlorodiphenyltrichloroethane and Malathion. Its preimaginal development in wells and water tanks was susceptible to predation by the larvivorous guppy fish *Poecilia reticulata*. The arrival, establishment, and spread of *An. stephensi* in northern Sri Lanka are analyzed in relation to anthropogenic factors that favor its range expansion. The implications of the findings for global public health challenges posed by malaria and other mosquito-borne diseases are discussed.

## Introduction

*Anopheles stephensi* is the most important vector of malaria in urban areas of India, and is present as three major biotypes *viz*. *type, intermediate* and *mysorensis* (1). The *type* form, also referred to as *An. stephensi sensu stricto*, and the *intermediate* form are mainly responsible for transmitting malaria in urban and rural areas, respectively, of India. The *mysorensis* form is considered to be a poor malaria vector because of its primary zoophagic nature (1).

**Figure 1:**
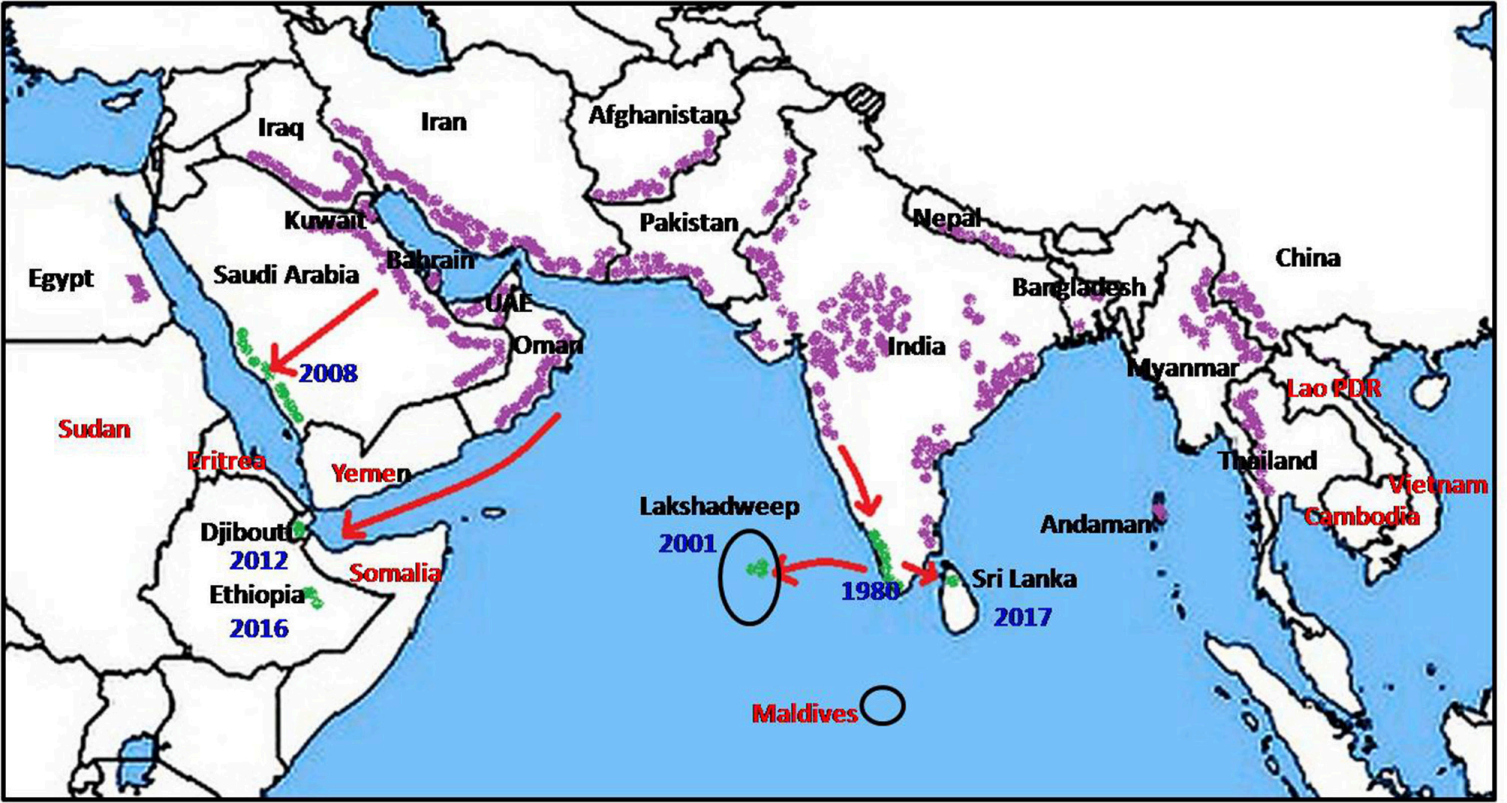
Global prevalence of *Anopheles stephensi* (purple), recent invasion to new countries (green) and countries at risk (red).

*Anopheles stephensi*, first described by Liston in 1901 at Ellichpur in the Maharashtra state of India (2) is now known to be present in large tracts of Asia and the Middle East (Figure 1). Its documented range extends from Egypt, Iraq, Iran, Saudi Arabia, Bahrain, Kuwait, United Arab Emirates, and Oman in the West through Afghanistan, Pakistan, India, Nepal, and Bangladesh in the Indian subcontinent, to Myanmar, South China, Thailand, and Vietnam in the East (1, 3–12).

Nation-wide entomological surveys initiated in 1975 in Kuwait did not record any anopheline larvae until 1981 when *An. stephensi* larvae were first identified (7). The population of *An. stephensi* in Kuwait underwent preimaginal development in farms bordering urban areas and within the capital city (7). The presence of *An. stephensi* in the eastern Saudi Arabia was first reported in 1956, in the Riyadh region in 2007 (13) and subsequently in the western region in 2008 (14). The expansion of mosquito fauna including *An. stephensi* in Saudi Arabia was attributed to the favorable conditions created by rapid social development and urbanization (11). Two further westward range expansion (Figure 1) occurred in recent times, possibly originating from *An. stephensi*-native territories bordering the Persian Gulf, via the Gulf of Aden to Djibouti in 2013 (15) and Ethiopia in 2016 (16). The invasion into Djibouti was postulated to have been caused by transportation of goods and movement of refugees returning from Oman (15). Such rapid range expansion now opens the possibility that *An. stephensi* may soon invade neighboring countries such as Somalia, Eritrea and Sudan.

**Figure 2:**
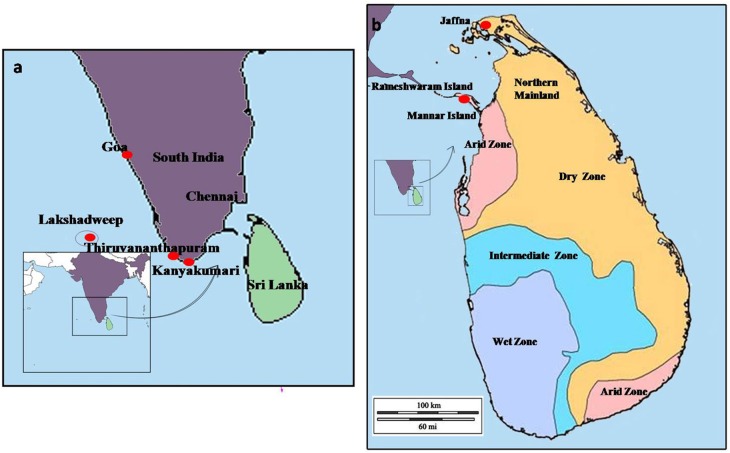
The map of **(a)** the island of Sri Lanka in the Indian Ocean, and **(b)** sampling sites in Mannar and Jaffna. The solid red circles indicate the southward expansion of *An. stephensi* into South India, Lakshadweep islands, and Sri Lanka.

A southward range expansion of *An. stephensi*, and attendant malaria transmission, was observed in India in the latter half of the Twentieth century and attributed to urbanization and associated water storage practices (17). As shown in Figure 2, *An. stephensi* reached Goa in the 1970s, Kanyakumari at the southernmost location of India in the 1980s, and subsequently Lakshadweep islands in the Arabian sea in 2001 (17). It was predicted in 2001 that *An. stephensi* may further expand southwards to invade Sri Lanka and the Maldives (17). *Anopheles stephensi* was detected for the first time in Sri Lanka in 2017 in the island of Mannar along the northwestern coast of Sri Lanka which is in close proximity to India (18, 19).

Malaria had been endemic in Sri Lanka for centuries but was eliminated from the island in 2013 due to efforts that employed vector control as the principal strategy (20). Malaria had previously been transmitted in Sri Lanka by *Anopheles culicifacies* species E as the primary vector, *An. annularis, An. subpictus* and *An. sundaicus* as important secondary vectors, and *An. vagus, An. aconitus, An. nigerrimus, An. pallidus* and *An. peditaeniatus* as minor vectors (21–29). These studies and comprehensive and regular entomological surveys carried out by the Anti-Malaria Campaign (AMC) of the Ministry of Health had never detected *An. stephensi* in the island prior to 2017. Malaria was mainly confined to rural areas in the dry and intermediate rainfall zones of Sri Lanka (Figure 2), and linked with clear fresh water collections associated with the riverine systems of the country which are the preferred habitat for the major malaria vector *An. culicifacies* (29). However, *An. culicifacies* was more recently shown to have adapted to develop in brackish water in eastern Sri Lanka (30) and the northern Jaffna peninsula (31). Fresh water is defined as containing <0.5 parts per thousand or ppt salt, brackish water between 0.5and 30 ppt salt, and saline water as containing >30.0 ppt salt (32).

The insecticide dichlorodiphenyltrichloroethane or DDT was introduced as an indoor residual spray (IRS) for malaria vector control after World War 2 in Sri Lanka, but a serious malaria epidemic occurred in 1967–1970 due to the development of resistance to DDT in *An. culicifacies* and possibly other vectors (20). The AMC began replacing DDT with the organophosphate Malathion for IRS in the early 1970s, but changed to pyrethroids in the early 1990s because the major mosquito vectors had also begun developing resistance to Malathion (26) leading to epidemics in 1985–1988 and 1991–1994 (20). Pyrethroid-treated bed nets were also introduced as a supplementary malaria control measure in the 1990s (20).

Although Sri Lanka was officially declared to be free of malaria by the WHO in 2016, potential anopheline vectors are abundant in the island and remain capable of transmitting malaria from the many infected persons returning to or visiting the island from malaria-endemic countries (20). The detection of *An. stephensi sensu stricto* (*type* form), an efficient and major urban malaria vector in the India subcontinent, in Mannar island in 2017 adds to the difficulty of maintaining Sri Lanka free from malaria (18, 19). DNA sequences of the mitochondrial cytochrome oxidase subunit 1 gene (*cox1*) (18, 19), cytochrome b gene (*cytb*) and the internally transcribed spacer 2 of ribosomal RNA (ITS2) (19) showed that the *An. stephensi* collected at Pesalai in Mannar island were closely related to *An. stephensi* from South India. The biotype of the Pesalai isolates was established as *An. stephensi sensu stricto* or *type* form based on the spiracular index (19).

Mosquito vectors can rapidly evolve to develop in new habitats within their normal range as a result of anthropogenic activities and pressures, and it has been suggested that this in turn can facilitate range expansion and invasion of new territories (33). The adaptation of *An. stephensi* to lay eggs and undergo preimaginal development in domestic wells and cement water storage tanks in urban areas in India has been considered to facilitate range expansion into new territories (34, 35). We report here for the first time on the presence of *An. stephensi* in the densely populated Jaffna municipal area in the Jaffna peninsula of North Sri Lanka, a location that also lies in close proximity to the state of Tamil Nadu in mainland India (Figure 2), as well as the relevant biological and genetic characteristics of Jaffna and additional Mannar isolates of *An. stephensi*. We also discuss the relevance of these findings in the context of range expansion in *An. stephensi* and other mosquito vectors and the global control of mosquito-borne diseases.

## Methods

### Larval Collections

**Figure 3:**
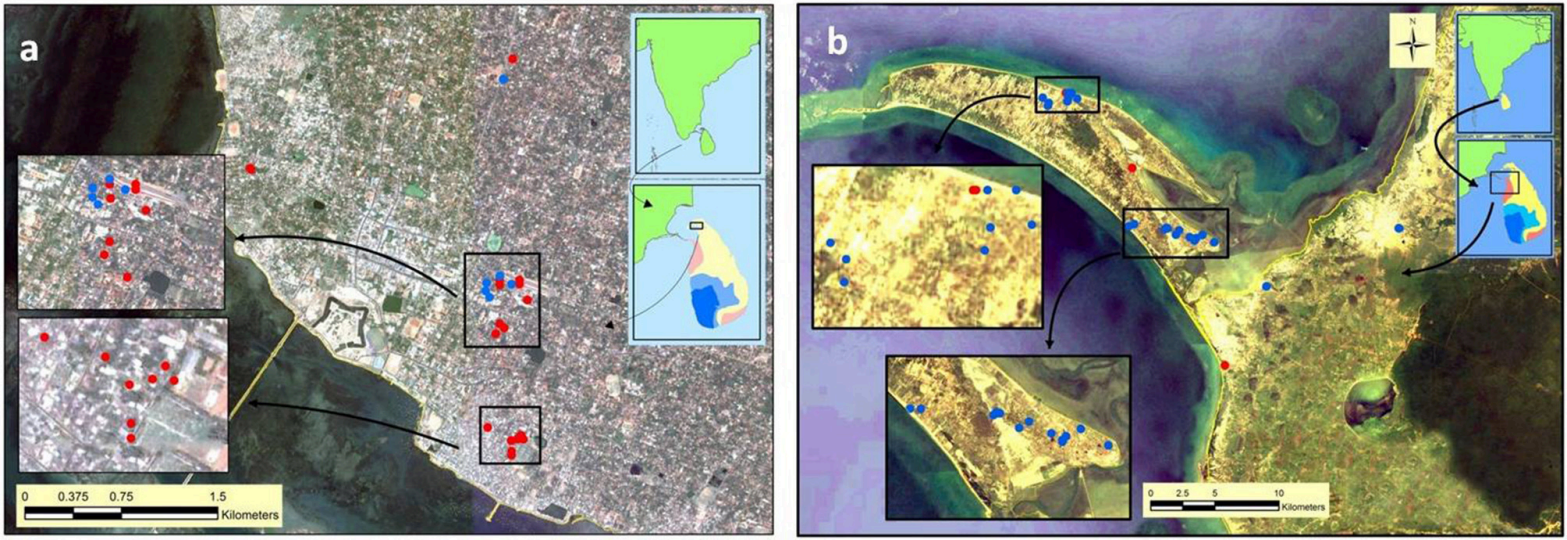
Map showing sites surveyed for anopheline larvae in **(a)** Jaffna and **(b)** Mannar. Red and blue solid circles, respectively, show *An. stephensi* positive and negative sites in the two locations.

Larvae were collected from several sites in the Jaffna Municipal Area (together termed Jaffna) as well as Mannar Island and Vankalai, a site adjoining Mannar island on the mainland, (together termed Mannar) as shown in Figure 3. Collections were done in Mannar on 29 September and 3 October 2017 and in Jaffna on 9, 10, and 12 October 2017. Mannar is located in the arid zone of Sri Lanka that receives an average annual rainfall of <800 mm, and Jaffna in the country's dry zone which receives average annual rainfall of between 800 and 1,900 mm (19). The two locations, approximately 96 km apart, are in close proximity to the state of Tamil Nadu in South India (Figures 2, 3). The island of Sri Lanka also has a wet zone that receives > 1,900 mm of annual rainfall and an intermediate rainfall zone with mixed dry and wet zone characteristics (Figure 2). Open domestic wells used to draw water for household purposes, as well as open, above ground cement tanks utilized to store water for household use were surveyed for anopheline larvae during the study.

Anopheline larvae were collected from deep wells using string-connected conical drop nets 15 cm diameter and 10 cm deep. Standard dippers (350 ml) were used to collect larvae from water storage tanks and shallow wells with 6 to 10 dips in each tank or well. Collected larvae were reared as described previously under contained conditions in the insectary of the Department of Zoology, University of Jaffna to reach adulthood (31). Larvae were maintained under laboratory conditions (28 ± 2°C, 12 h photoperiodicity and ~ 70% relative humidity) in the same water from the habitats where they were collected in 1.5 L capacity plastic trays with powered fish meal given twice a day as additional food. The emergent adults were identified morphologically as *An. stephensi* using a published key (36, 37) and then used for characterization of biotype and genotype.

The Jaffna regional AMC introduced the larvivorus guppy fish *Poecilia reticulata* in the period 12 to 17 October 2017 to all the domestic wells and water tanks that our surveys had shown to contain *An. stephensi* larvae. Three to five pairs of fish were introduced depending on the size of the habitat. Larval surveys were therefore continued from 20 October 2017 to 01 February 2018 in these wells and water tanks in order to examine the effect of introducing the guppy on larval prevalence.

### Determination of Salinity

Water samples were taken to the laboratory where salinity was determined using a Hach HQ30d single channel multiparameter meter (Hach, UK).

### Determination of *Anopheles Stephensi* Biotype Based on Egg Morphology and Spiracular Index

Newly emerged adult males (~25) and females (~20) of *An. stephensi* larvae collected from sites in Mannar were fed on sugar pledgets and on the fifth day allowed to blood-feed on Balb/c mice. Blood-fed females were kept individually in small plastic cups with egg laying surfaces as described previously (38). Five to eight eggs from each female were placed on a microscopic slide and the number of ridges on floats counted under the 4x objective in an Olympus light microscope. Adult *An. stephensi* originating from larvae collected in Jaffna and Mannar were used for determining their biotype based on the spiracular index as described previously (19). The calculated spiracular indices were subjected to a two-tailed Student's *t*-test to identify significant differences in their means.

### Insecticide Susceptibility Tests

Morphologically identified adult *An. stephensi* F_0_ generation female mosquitoes emerging from larvae collected in Jaffna and their F_1_ and F_2_ progeny were exposed to insecticide impregnated papers of 0.05% (w/v) deltamethrin, 5% (w/v) Malathion and 4% (w/v) DDT using WHO bioassay kits and methodology (39). Mosquitoes in batches of 10–15 were exposed for 1 h and the mortalities determined after a 24 h recovery period. Five to six replicate tests were done with each insecticide. Control mosquitoes were exposed to papers impregnated with carrier oil alone. A total of 100 to 120 adults were exposed to each insecticide. The susceptibility was assessed and mortality corrected using Abbot's formula (40).

### DNA Sequencing of the *Cox1* Gene and Population Genetic and Phylogenetic Analysis

DNA from 15 individuals each from the Jaffna and Mannar collections, and morphologically identified as *An. stephensi*, was extracted using the DNeasy Blood & Tissue kit (Qiagen, CA, USA). A region of the *cox1* gene was amplified by using primers C1-J-1718 and C1-N-2191 (41). For each amplification, PCR reactions were performed in a 25 μl volume as described previously (42). The PCR products were purified using QIAquick® PCR Purification Kit (Qiagen, CA, USA). Purified PCR products of were sequenced in both directions at Macrogen, South Korea.

The chromatograms of *cox1* sequences were manually edited using BioEdit software (43) and consensus sequences were aligned along with available South Indian sequences from the states of Tamil Nadu and Puducherry for *An. stephensi* retrieved from GenBank using Clustal W in MEGA 5.0 software (44). The aligned sequences for coding genes were translated into amino acid sequences using the invertebrate mitochondrial codon usage pattern. Number of haplotypes, genetic diversity indices [Haplotype Diversity Index (Hd) and Nucleotide Diversity Index (Pi)] and Neutrality tests (Tajima's *D* and Fu's *F*s) were performed in DNA sequence polymorphism software (DNASP) Version 5.1.10 (45). Pairwise difference due to genetic structure (Fst) of Jaffna, Mannar and Tamil Nadu/Puducherry populations was analyzed in Arlequin 3.11 version and the significance was evaluated based on 1,000 permutations. *Cox1* sequence-based maximum likelihood phylogenetic analysis was performed using the Tamura 3-parameter. One thousand nonparametric bootstrap replicates were done and a consensus tree was constructed in MEGA 5.0 software (44).

## Results

### *Anopheles Stephensi* Larval Collections in Jaffna and Mannar

*Anopheles* larvae could be collected during the present study in Jaffna (a total of 201 larvae) and Mannar (a total of 51 larvae). Of the adults emerging from the 252 larvae, 201 were identified as *An. stephensi* and all others as *Anopheles varuna*. Details of the sites where *An. stephensi* were collected are shown in Figure 3 and Table 1. One of the *An. stephensi* larvae-positive sites in the present study in Mannar was Pesalai, which is a fishing settlement where *An. stephensi* was first identified during a preceding study (19). The nature of the larval habitats of *An. stephensi* at the different sites and the range of salinity observed in the habitats in Jaffna and Mannar are shown in Table 1.

**Table 1:** *Anopheles stephensi* presence and salinity in surveyed larval habitats in Mannar and Jaffna.

**Location**	**Site**	**GPS coordinates**	**Number of potential habitats inspected**	**Habitat type**	**Number of habitats with *An. stephensi* larvae (salinity range in ppt salt)**	**Number of larvae identified as *An. stephensi***
Mannar	Pesalai	9°05′11″N 79°49′11″E	4	Wells	2 (0.5, 0.7)	19
	Chinnakadai	8°58′59″N 79°54′36″E	7	Wells	0	–
	Thottaveli	9°02′02″ N 79°51′59″ E	6	Wells	1 (0.7)	8
	Pallimunai	8°58′55″N 79°55′26″E	5	Wells	0	–
	Thrukethisvaram	8°57′04″N 79°57′38″E	2	Wells	0	–
	Vankalai	8°53′45″N 79°55′53″E	3	Cement tanks	1 (0.4)	12
**Total for Mannar**			**27**	**Total for Mannar**	**4**	**39**
Jaffna	Jaffna town	9°39′57″N 80°01′10″E	13	Wells	8 (0.8–3.0)	63
	Gurunagar	9°39′13″N 80°01′13″E	8	Wells/cement tanks	8 (1.6–3.5)	68
	Navanthurai	9°40′23″N 80°01′13″E	2	Wells	2 (1.2, 2.3)	19
	Thirunelvely	9°40′51″N 80°01′10″E	2	Wells	1 (0.6)	12
**Total for Jaffna**			**25**	**Total for Jaffna**	**19**	**162**

In the period 9–12 October 2017, *An. stephensi* larvae were found in 19 of the 25 wells and water tanks examined in Jaffna. However, after *P. reticulata* was introduced in mid-October 2017 by the Jaffna regional AMC, only 9 of the same 25 habitats remained positive for *An. stephensi* larvae on 20 October 2017. Larvae were not found in the same 25 wells and tanks in subsequent surveys done on 3 November 2017, 25 January 2017, and 1 February 2018.

### Determination of Biotype

Twenty-two and 18 adults from the Jaffna and Mannar collections respectively were used for determining biotype of *An. stephensi* based on the spiracular index. The average spiracular length varied between 0.09–0.13 mm (0.11 ± 0.01, mean ± standard deviation) and 0.09–0.12 mm (0.11 ± 0.01), and the thoracic length varied between 0.92–1.32 mm (1.12 ± 0.08) and 1.06–1.24 mm (1.14 ± 0.07) for the Jaffna and Mannar samples, respectively. The calculated spiracular index varied from 8.02–11.32 (9.25 ± 0.75) to 8.75–10.38 (9.39 ± 0.48), respectively, for the Jaffna and Mannar *An. stephensi*. The calculated spiracular index for the Jaffna *An. stephensi* was not significantly different from that for Mannar *An. stephensi* samples [*t*_(38)_ = 0.686, *P* = 0.497]. The number of egg ridges in 64 eggs from 10 iso-females of Mannar collection varied from 15 to 18 (16.2 ± 1.0). A comparison with published biotype data for the spiracular index (46) and number of egg ridges (47, 48) in India showed that both Mannar and Jaffna populations belong to the urban biotype *An. stephensi sensu stricto* or *type* form identified in India.

### Susceptibility to Insecticides

The Jaffna population of *An. stephensi* showed 15 ±4% (mean± standard deviation), 55 ± 15% and 64 ± 3% susceptibility to 4% DDT, 5%, Malathion and 0.05% deltamethrin, respectively. Since they were <80% susceptible, they were classified as being resistant to all three insecticides (39).

### *Cox1* Gene Sequence and Population Genetic and Phylogenetic Analysis

After trimming the *cox1* sequences to remove ambiguous ends, final fragments of 467 bp of 9 and 11 isolates from Mannar and Jaffna, respectively, were used in the genetic diversity and population genetic structure analysis. The *cox1* sequences were free of nuclear gene copies and were consistent with the reference genome of *An. stephensi*. No deletions, insertions or stop codons were observed. This indicated the absence of pseudogenes. Translated amino acid sequences did not show frame shifts or stop codons. All analyzed sequences were overlapping and covered the same region. The single nucleotide variation resulted in a transition where a G [haplotype H1; GenBank accession numbers: MG970564 (Jaffna) and MG970566 (Mannar)] is replaced by an A [haplotype H3; GenBank accession number: MG970565 (Jaffna) and MG970567 (Mannar)] and previously reported for Mannar samples (19) was also present in the Jaffna and Mannar populations of *An. stephensi* collected in the present study. This nucleotide variation causes a change in the amino acid sequence from valine to methionine in the *cox1* protein. Only two haplotypes were found in the Jaffna and Mannar *An. stephensi* populations. The calculated haplotype diversities (Hd) and nucleotide diversities (Pi) of the two populations are shown in Table 2.

**Table 2:** Genetic diversity indices and neutrality tests for *An. stephensi* populations from Mannar and Jaffna compared with Tamil Nadu and Puducherry in South India.

**Population** ** (number of sequences analyzed)**	**S**	**Number of sequences for each haplotype**	**Hd (± sd)**	**Pi (± sd)**	**D**	**Fs**	**Reference GenBank accession number for haplotypes**	**F_ST_**	
								**Jaffna**	**Tamil Nadu /Puducherry**
Mannar (9)	1	H1 = 4 and H3 = 5	0.389 ± 0.164	0.0011 ± 0.001	0.155 (*P* > 0.1)	0.477 (*P* > 0.1)	MG975066 MG975067	0.474 *P* < 0.05	0.598 *P* < 0.05
Jaffna (11)	1	H1 = 7 and H3 = 4	0.327 ± 0.153	0.0009 ± 0.001	−0.100 (*P* > 0.1)	0.356 (*P* > 0.1)	MG970564 MG970565		0.061 *P* > 0.25
Tamil Nadu/Puducherry (6)	1	H1 = 5 and H2 = 1	0.333 ± 0.215	0.00091 ± 0.001	−0.933 (*P* > 0.1)	−0.003 (*P* > 0.1)	DQ317594 DQ310148		

*S, number of polymorphic sites; Hd, haplotype diversity; Pi, nucleotide diversity; D, Tajima's D; Fs, Fu's Fs; F_ST_, fixation index; sd, standard deviation of mean*.

**Figure 4:**
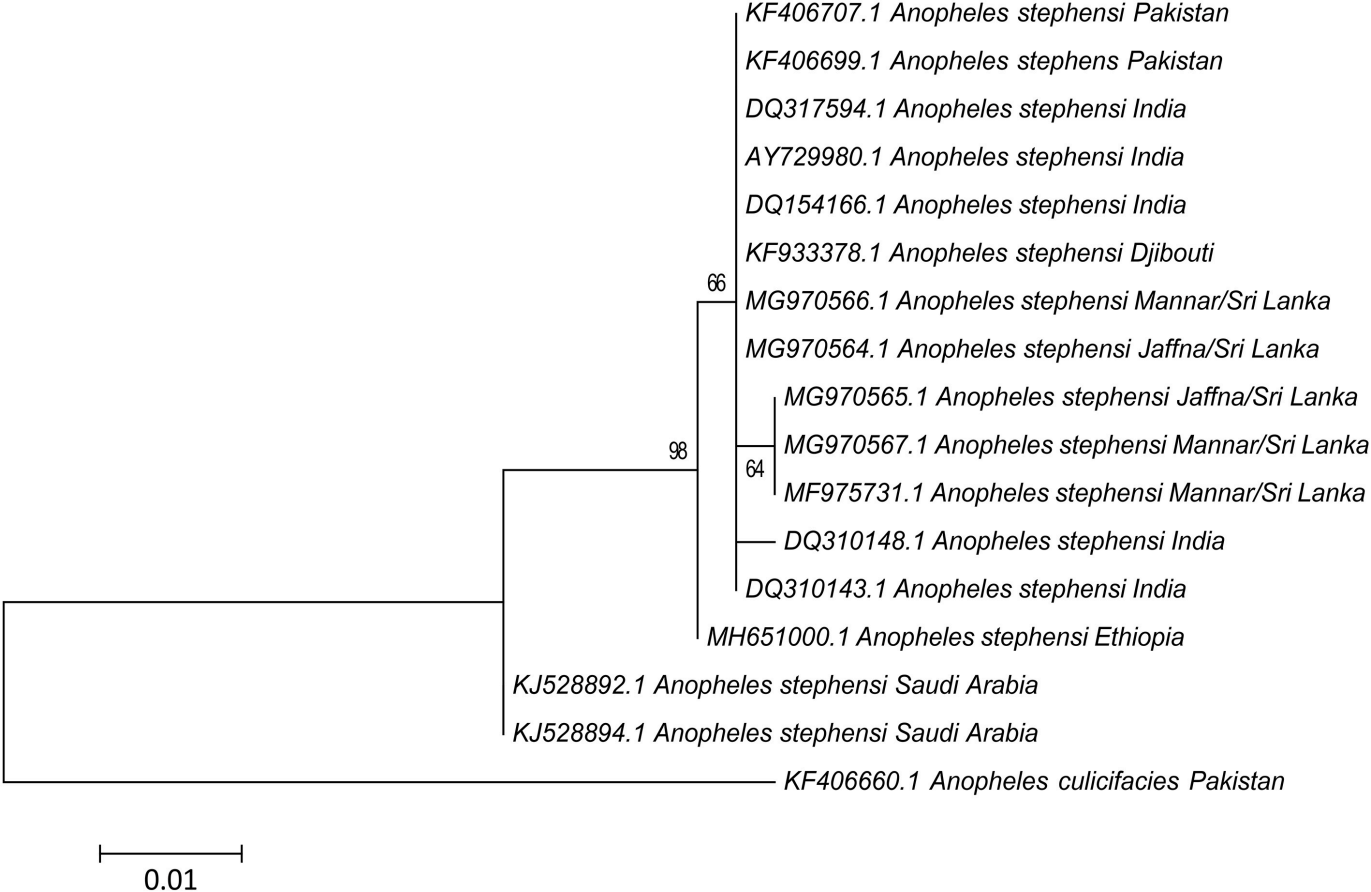
Phylogenetic analysis based on *cox1* sequences (367 nucleotide positions) constructed using the maximum likelihood method and Tamura 3-parameters showing bootstrap values > 60%. The sequences used for analysis include samples from Jaffna (GenBank: MG970564 and MG970565), Mannar (GenBank: MG970566 and MG970567) described in this study and other available GenBank entries. *Anopheles culicifacies* (GenBank: KF406660) was used as the outgroup.

According to neutrality test results both Tajima's *D* and Fu's Fs values were not significant (*p* > 0.1) in the Mannar and Jaffna populations (Table 2). The most dominant haplotype H1 is shared by the two Sri Lankan populations and samples from South India. Haplotype H3 is only present in the two Sri Lanka populations and more commonly in the Mannar populations. Haplotype H2 GenBank accession number DQ310148 is only present in Tamil Nadu and Puducherry in South India (Table 2). The pairwise comparison of population differentiation revealed that the Jaffna population tended to be genetically closer to the known South Indian population (Fst = 0.061; *P* > 0.25), whereas the Mannar population was significantly genetically different from the Jaffna (Fst = 0.474; *P* < 0.006) and known South Indian (Fst = 0.597; *P* < 0.006) populations. The phylogeny trees created using the available *cox1* sequence data is presented in Figure 4. As reported previously (19) only one base difference was present within the Sri Lankan group for *cox1*. The other *cox1* sequences, except samples from Saudi Arabia, did not vary enough to create separate clades with strong bootstrap values.

## Discussion

The present study showed that the urban malaria vector *An. stephensi sensu stricto* (*type* form) has established itself in two coastal locations in northern Sri Lanka *viz*. Jaffna and Mannar that are approximately 96 km apart. Both spiracular index and, for the first time, the number of ridges in eggs were be used to determine the presence of the *type* form in Sri Lanka. *Anopheles stephensi sensu stricto* or *type* form is the dominant vector of urban malaria in India (1, 34, 35). The sites where *An. stephensi* larvae were found in Jaffna and Mannar have urban or semi-urban characteristics.

*Anopheles stephensi* has evolved to adapt to both rural and urban environments in India (1). The rural *An. stephensi* populations in India (*An. stephensi mysorensis* and *An. stephensi intermediate* forms) develop in freshwater ponds, stream beds, wells and seepage canals while the urban *An. stephensi sensu stricto* or *type* form populations develop mainly in artificial water containers, over ground water storage tanks, domestic wells, underground cement water storage tanks, evaporator coolers, cisterns, barrels, roof gutters, pits in construction sites and ornamental tanks (1, 49, 50). The present findings show that the Jaffna and Mannar populations of *An. stephensi sensu stricto* are able to lay eggs and undergo preimaginal development in man-made wells and cement water storage tanks that also constitute their urban habitats in India. Other potential urban and rural habitats were not surveyed, and adult *An. stephensi* mosquito collections were not attempted during this study due to the limited resources available, but are important as follow-up studies for a more complete understanding of the establishment of *An. stephensi* in Sri Lanka.

The present results also show that *An. stephensi sensu stricto* can lay eggs and undergo preimaginal development in brackish water of up to 3.5 ppt salt in Jaffna and 0.7 ppt salt in Mannar. Previous studies have shown that *An. stephensi* has euryhaline characteristics and is able to develop in brackish water in Pakistan (51, 52). Anthropogenic environmental modifications that caused water logging and salinization favored salinity-tolerant *An. stephensi* (51) becoming the more dominant malaria vector over *An. culicifacies* in South Punjab, Pakistan (53). The northern Jaffna peninsula has a limestone geology and salinization of fresh water aquifers due to the incursion of sea water is an increasing problem (54). Brackish water collections in beach debris, fishing boats and domestic wells have been shown to provide habitats for the preimaginal development of *Aedes aegypti, Aedes albopictus, An. culicifacies* and other anopheline malaria vectors in coastal areas of North and East Sri Lanka (28–32, 54, 55). It is therefore likely that *An. stephensi sensu stricto* that has evolved to undergo preimaginal development in brackish water, can utilize similar anthropogenic brackish water habitats to further extend its range within the Jaffna peninsula and other coastal areas of Sri Lanka. Rising sea levels resulting from global warming will increase saline intrusion and expand the extent of brackish water bodies in coastal areas that in turn can facilitate salinity adaptation in fresh water vectors and increase the numbers of existing salinity-tolerant vectors (56). The impact on vector-borne disease transmission can be considerable because a large proportion of the world's population live coastal zones that are liable to increasing salinization (54, 56–58).

Our results also show that the introduction of the larvivorus guppy fish *P. reticulata* into domestic wells was effective in eliminating *An. stephensi* larval development in the surveyed wells in Jaffna. We previously demonstrated the ability of the tilapia fish *Oreochromis mossambicus* to effectively in control *Aedes* and *Anopheles* larvae in water storage tanks in Jaffna (59). Therefore, the introduction of both species of larvivorous fish can constitute an important adjunct to other efforts to the control or eliminate urban *An. stephensi* in Sri Lanka.

The Jaffna population of *An. stephensi* is resistant to DDT, Malathion and deltamethrin. The three insecticides have been widely used for IRS in recent times in Sri Lanka. There is continuing use of organophosphates related to Malathion in agriculture and pyrethroids related to deltamethrin for personal protection and agriculture in the Jaffna peninsula. Malaria control in urban India also relies heavily on larval control and personal protective measures using insecticides impregnated nets and repellents (60). Pyrethroids are the major constituents of insecticide impregnated nets and mosquito repellent devices such as coils, evaporators and mats which are commonly used in urban areas in India and Sri Lanka (20, 60). Resistance, partial resistance or susceptibility to DDT, Malathion and deltamethrin in *An. stephensi* populations have variously been reported recently at different locations in India but there is no published data from coastal areas of Tamil Nadu that are proximal to northern Sri Lanka (60–62). It is possible that the development of resistance to Malathion and deltamethrin in the Jaffna *An. stephensi* population may be an indigenous phenomenon but there is not enough data to show that such resistance did not originate in the neighboring coastal districts of Tamil Nadu. It was not possible to investigate the biochemical and genetic basis for resistance to insecticides in the Jaffna *An. stephensi* due to resource limitation but this merits further investigation and a comparison with *An. stephensi* populations in neighboring areas of Tamil Nadu and elsewhere in Sri Lanka. Although the insecticide resistance seen in *An. stephensi* is likely to be associated with a fitness cost (63), it can facilitate the survival and spread of the new invasive species in Jaffna and elsewhere in Sri Lanka.

The population genetic analysis shows that the both Jaffna and Mannar *An. stephensi* populations share genetic similarity with Tamil Nadu and Puducherry populations even though Mannar populations show significant genetic structuring from both the Jaffna and Tamil Nadu/Puducherry populations. Significant population genetic structuring observed between Mannar and Jaffna *An. stephensi* populations cannot be easily attributable to a distinct geographical barrier as Mannar is connected to Jaffna by a good road network and a causeway. The haplotypes (H3) that shows the point mutation for a G to A transition that results in an amino acid change from valine to methionine appears to be more prevalent in Mannar than Jaffna. It has only been reported in Sri Lankan *An. stephensi* to date in the limited number of sequences deposited in GenBank. Presence of a selection pressure in a population is reflected by neutrality tests. The results of neutrality tests and Tajima's *D* and Fu's Fs of *cox1* data were not significant and this is an indication that there is no selection pressure for any of the identified haplotypes in the Jaffna and Mannar *An. stephensi* populations.

Biological invasion or range expansion into new territories may be regarded to occur in three phases *viz*. arrival, establishment and spread (64, 65). The spread of *An. stephensi sensu stricto* into Sri Lanka, very likely from the Indian state of Tamil Nadu due to proximity and genotype data, may be analyzed in this framework.

### Arrival

The movement of people has been an important factor in the geographic spread of insects to new territories (66). For example, whole genome sequencing of the arboviral vector *Aedes aegypti* strongly suggests that this mosquito spread within a few centuries from an origin in West Africa to colonize Asia and Central America as a result of human migration (67). The movement of refugees and combatants between Tamil Nadu and northern Sri Lanka was a characteristic of the 1983–2009 civil war. Also fishermen have traditionally moved relatively freely across the 64 to 137 km-wide Palk strait that separates northern Sri Lanka from Tamil Nadu. Malaria control efforts and IRS as well as mosquito surveys were severely disrupted in northern Sri Lanka during the 1983–2009 civil war. Because comprehensive and regular surveys by the AMC as well as academic studies (21–28) had not shown the presence of *An. stephensi* in Sri Lanka prior to 2017, it is reasonable to assume that *An. stephensi* arrived in Sri Lanka from Tamil Nadu sometime during the civil war of 1983–2009. Similarly, the spread of *An. stephensi* into Djibouti has been attributed to the movement of refugees from Oman across the Gulf of Aden (15). While the transport of gravid female *An. stephensi* in boats is a possibility, it seems more likely that eggs and larvae in brackish water collections within small fishing boats, established to be a habitat for salinity-tolerant *Ae. aegypti* larvae in Jaffna (32), was the probable method of arrival.

There is not enough data to determine whether there were one or more than one spatially distinct arrivals of *An. stephensi* in northern Sri Lanka. It is possible that the Jaffna and Mannar populations of *An. stephensi* originated from a single arrival from Tamil Nadu sharing the haplotypes H1 that then underwent dispersion and micro-evolution to produce the haplotypes H3 –a haplotype which has not yet been reported in the small numbers of South Indian *An. stephensi* that have been genotyped to date. An alternative, and more likely possibility, is that the Jaffna and Mannar populations are derived from two or more separate founding events each involving a small number of larvae or eggs originating in Tamil Nadu.

### Establishment

*Anopheles stephensi* may be regarded to have an origin as a rural mosquito vector that evolved into the *An. stephensi sensu stricto* or *type* form on its adaptation to lay eggs and develop in man-made sites such as domestic wells and cement water storage tanks, causing the *An. stephensi sensu stricto* or *type* form to become the major urban malaria vector in the Indian subcontinent. Rapidly increasing population with associated urbanization that expanded the numbers of domestic wells and water storage containers in the Indian subcontinent was probably an enabling factor in this evolutionary process (35). Anthropogenic changes to the environment are known to exert selection pressure on phenotypes in a variety of taxa (68, 69). In particular, urbanization can modify and fragment habitats and thereby lead to phenotypic trait selection in different taxa (69), including mosquito vectors (33). The long term effect of urbanization in North America on mosquito populations has been well documented (70). The adaptation of *Ae. aegypti* to from a forest dwelling, zoophagic form to an urban anthropophagic form in Africa has been related to the evolution of an odorant receptor that recognizes a human odor component (71). The subsequent spread of *Ae.aegypti* worldwide is a likely to have been facilitated by such adaptation to anthropophagicity and attendant co-adaptation to develop in the vicinity of human habitations (67). The evolution of the *An. stephensi sensu stricto* or *type* from the possible ancestral *mysorensis* form may have followed a similar path. Urban malaria transmitted by *An. stephensi sensu stricto* was first recognized in 1969 in India (34, 35), leading Indian authorities to formulate an Urban Malaria Scheme in 1971–72 (35). Domestic wells and cement water storage tanks are common in the densely populated Jaffna city and the rest of the peninsula because the availability of piped water drawn from artesian wells is restricted to Jaffna city and then only for certain times of the day. Therefore, three factors may be considered to be important for enabling *An. stephensi sensu stricto* to establish itself in Mannar and Jaffna after arriving from Tamil Nadu, India. These are (i) the readily availability of urban domestic wells and cement water storage tanks as habitats to which it was already adapted in India, (ii) the adaptive evolution to lay eggs and undergo preimaginal development in brackish water man-made habitats in coastal areas which may also have been previously acquired in coastal areas of Tamil Nadu, and (iii) resistance to pyrethroid, organophosphate and organochlorine insecticides which may also have been previously developed in Tamil Nadu. The three factors are examples of what was previously termed the anthropogenically-induced adaptation to invade in mosquito vectors of human disease (33).

### Spread

The results demonstrate the spread of *An. stephensi* to different sites within Mannar and Jaffna in Sri Lanka. As discussed above it is not clear whether the Mannar and Jaffna populations are a result of spread along the northwestern coast from a single arrival event or are the result of multiple arrivals. Only the AMC has the resources to perform surveys to detect *An. stephensi* over large tracts of the northern coast and indeed the rest of the island that are required to determine the extent of its spread in Sri Lanka, understand the number of arrival events from Tamil Nadu and study the proposed mechanisms underlying the spread. However, it is reasonable to speculate that the three factors (i) to (iii) above facilitating the establishment of *An. stephensi sensu stricto* in Mannar and Jaffna are also likely to be important factors that govern its spread elsewhere in the country.

In conclusion, the recent spread of *An. stephensi* to new territories in Ethiopia, Djibouti, Lakshadweep islands are also likely to have been caused by anthropogenic and urbanization-associated factors similar to those discussed for Sri Lanka. Expansion of *An. stephensi* to the Sudan, Eretria and Somalia in the West; Laos, Cambodia and Vietnam in the East; and Maldives in the South due to the same anthropogenic factors is a likely possibility and a cause for concern for the public health authorities (Figure 1). Broad appreciation of the effects of anthropogenic drivers of mosquito vector-adaptation by global health decision-makers, and the development of appropriate mitigating strategies is clearly important. The findings that larvivorous fish can eliminate *An. stephensi* in wells and water tanks however suggests one approach for controlling the spread of this potent vector of malaria.

## Ethics Statement

This work has been carried out as a part of an ongoing mosquito survey with the Approval (AERC/2017/02) of the Animal Ethics Committee of the University of Jaffna (AERC/UoJ). Protocol for feeding mosquitoes on animals was approved (AERC/2014/02) by the AERC/UoJ. Verbal informed consent was obtained from householders to collect larvae from their domestic wells and water storage tanks.

## Author Contributions

SNS and RR: conceived and designed the study and wrote the manuscript. AS, TJ, and KS: carried out the field studies. KS, KG, and MS: carried out the laboratory studies. SNS, AS, MS, and SK: performed the analysis. SK: contributed to the content of the manuscript. All authors read and approved the final manuscript.

### Conflict of Interest Statement

The authors declare that the research was conducted in the absence of any commercial or financial relationships that could be construed as a potential conflict of interest.
